# Comparison of Reported Spinal Cord Lesions in Progressive Multiple Sclerosis with Theiler’s Murine Encephalomyelitis Virus Induced Demyelinating Disease

**DOI:** 10.3390/ijms20040989

**Published:** 2019-02-25

**Authors:** Eva Leitzen, Wen Jin, Vanessa Herder, Andreas Beineke, Suliman Ahmed Elmarabet, Wolfgang Baumgärtner, Florian Hansmann

**Affiliations:** 1 Department of Pathology, University of Veterinary Medicine Hannover, 30559 Hannover, Germany; Eva.Leitzen@tiho-hannover.de (E.L.); Wen.Jin@tiho-hannover.de (W.J.); Vanessa.Herder@tiho-hannover.de (V.H.); Andreas.Beineke@tiho-hannover.de (A.B.); Kshty1@yahoo.com (S.A.E.); Florian.Hansmann@tiho-hannover.de (F.H.); 2 Center for Systems Neuroscience, 30559 Hannover, Germany

**Keywords:** axonal loss, multiple sclerosis, reticulospinal tract, spinal cord, spinal cord atrophy, Theiler’s murine encephalomyelitis virus

## Abstract

Background: Spinal cord (SC) lesions in Theiler’s murine encephalomyelitis virus induced demyelinating disease (TMEV-IDD) resemble important features of brain lesions in progressive multiple sclerosis (MS) including inflammation, demyelination, and axonal damage. The aim of the present study was a comparison of SC lesions in MS and TMEV-IDD focusing on spatial and temporal distribution of demyelination, inflammation, SC atrophy (SCA), and axonal degeneration/loss in major descending motor pathways. Methods: TMEV and mock-infected mice were investigated clinically once a week. SC tissue was collected at 42, 98, 147, and 196 days post infection, and investigated using hematoxylin and eosin (HE) staining, immunohistochemistry targeting myelin basic protein (demyelination), Mac3 (microglia/macrophages), phosphorylated neurofilaments (axonal damage) and transmission electron microscopy. Results: Demyelination prevailed in SC white matter in TMEV-IDD, contrasting a predominant gray matter involvement in MS. TMEV-infected mice revealed a significant loss of axons similar to MS. Ultrastructural analysis in TMEV-IDD revealed denuded axons, degenerative myelin changes, axonal degeneration, as well as remyelination. SCA is a consistent finding in the SC of MS patients and was also detected at a late time point in TMEV-IDD. Conclusion: This comparative study further indicates the suitability of TMEV-IDD as animal model also for the investigation of progressive SC lesions in MS.

## 1. Introduction

Multiple sclerosis (MS) is an autoimmune driven, inflammatory and degenerative disease of the central nervous system (CNS) characterized by inflammation, demyelination, as well as axonal damage and loss [1,2]. At present, it is the most common demyelinating disease affecting the CNS of young adults showing an increasing prevalence and incidence with approximately 2.5 million people affected worldwide [3,4]. It usually starts with relapsing and remitting episodes of impairment (relapsing remitting MS; RRMS) frequently converting into a progressive disease form with ongoing neurological decline (secondary progressive MS; SPMS), and fewer cases with a progression of neurological disability from onset (primary progressive MS; PPMS) [5,6]. The etiology of this currently incurable disease is still unknown but a complex interplay of genetic, infectious, environmental, and lifestyle factors contributing to the broad variability of disease phenotypes among MS patients is assumed [6,7,8,9,10]. MS lesions occur in brain and spinal cord (SC) but have almost exclusively been investigated in the brain [11,12,13,14,15]. Damage and loss of axons are key features of chronic disability especially in progressive MS [16,17,18]. Another finding strongly correlated with clinical disability in MS is spinal cord atrophy (SCA) [14,19,20,21]. SCA can occur as tissue loss in acute inflammatory SC lesions, but also occurs in the normal appearing white matter (NAWM) due to axonal degeneration [18,22]. MS brain lesions have been classified into four patterns according to cell types involved, demyelination, complement deposition, and axonal damage [12,23]. However, a similar classification for SC lesions is not available.

Different animal models are used for the investigation of MS pathogenesis and the development of new therapeutic strategies. However, pathomorphological similarities and differences between MS SC lesions and SC lesions in the animal models have not been investigated. Theiler’s murine encephalomyelitis virus (TMEV) belongs to the family of picornaviridae and represents an enteric pathogen in mice [24,25]. Following intracerebral infection of susceptible mice (e.g., SJL) with a low neurovirulent TMEV strain (e.g., BeAn), animals develop an acute polioencephalomyelitis followed by a chronic demyelinating leukomyelitis, characterized by inflammation, demyelination, and axonal damage [26,27,28]. This second phase, referred to as TMEV-induced demyelinating disease (TMEV-IDD), resembles important features of the progressive forms of MS and therefore serves as a suitable and frequently used animal model [25,26,27,28]. Imaging techniques such as magnetic resonance imaging (MRI) revealed an atrophy of brain and spinal cord in TMEV infected mice [29,30]. Brain atrophy precedes clinical signs whereas SCA occurs subsequent to clinical impairment at later time points [29]. Concomitantly with that, it has been stated that SCA in TMEV-IDD occurs following demyelination as a result of axonal loss [31,32]. On the other hand, it has also been hypothesized that axonal loss occurs as primary event independent of demyelination during the late phase of TMEV-IDD [33,34,35]. Similar pathomechanisms are discussed for axonal pathology in MS [36,37,38,39]. There are only few reports characterizing histological alterations of SC lesions in MS. The aim of the present study is to identify similarities and differences between SC lesions in TMEV-IDD and reported SC lesions in progressive MS. Special emphasis is given to SCA, inflammation, demyelination, and axonal damage to further substantiate the suitability of TMEV-IDD as a model for MS. 

## 2. Results

### 2.1. Clinic/Rotarod

**Figure 1:**
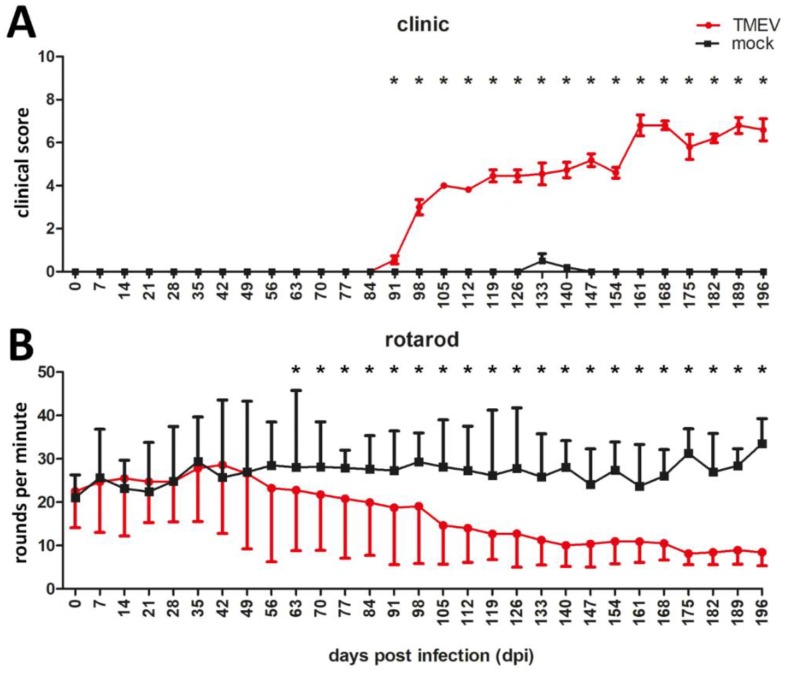
Clinical investigation and rotarod testing of TMEV- and mock-infected SJL mice. TMEV-infected mice showed significant clinical signs starting at 91 dpi (**A**) and a deterioration of motor coordination starting at 63 dpi (**B**) compared to mock infected animals. Graphs display mean and standard error of mean. Significant differences between the groups as detected by two-way repeated measure ANOVA with post-hoc independent t-tests for the different time points are marked by asterisks (* *p* ≤ 0.05).

Significant clinical signs were detected in TMEV infected animals starting at 91 days post infection (dpi) (Figure 1A). Rotarod data revealed a significant deterioration of motor performance starting at 63 dpi until the end of the investigation period (Figure 1B). Accordingly, evaluation of hematoxylin and eosin (HE) and/or Luxol fast blue (LFB) stained sections revealed foci of demyelination and inflammation, predominantly located within the ventral white matter areas of cervical and thoracic spinal cord at 42 dpi. Lesion size increased over time, affecting almost the entire ventral white matter and also the dorsal white matter in half of the TMEV-infected animals at 196 dpi. The lumbar spinal cord was less frequently affected and showed a timely-delayed lesion development compared with cervical and thoracic spinal cord. 

### 2.2. Area Measurement

**Figure 2:**
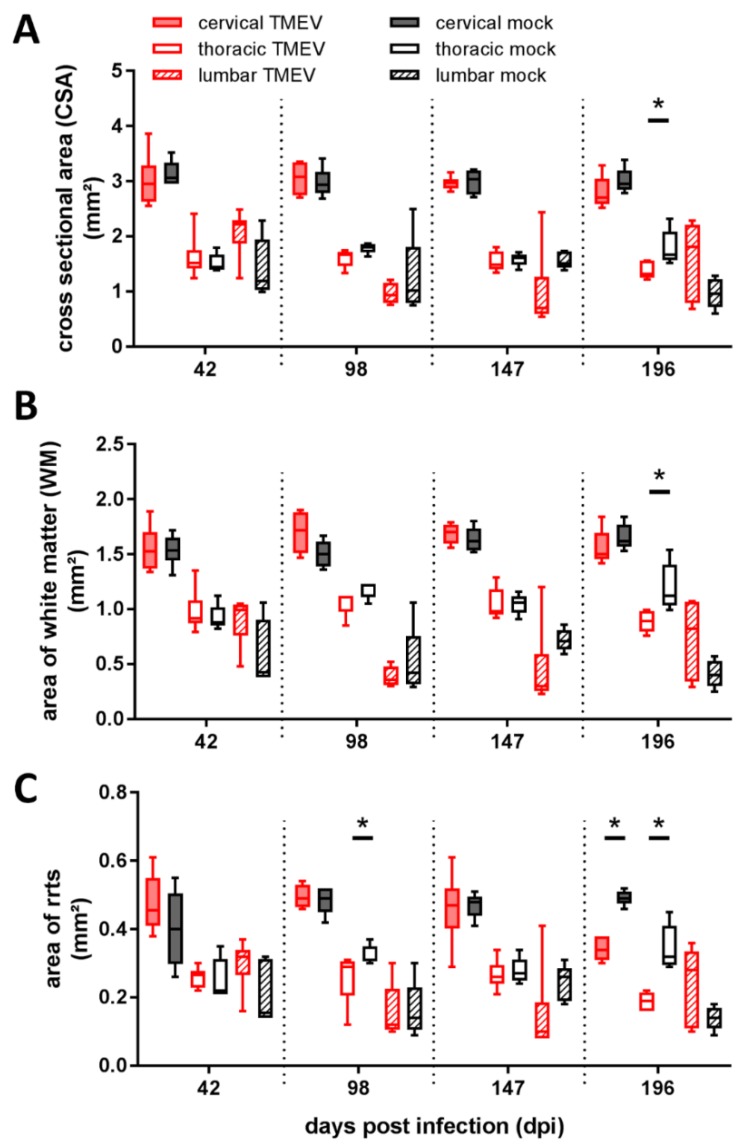
Assessment of spinal cord areas revealed a significant reduction of cross-sectional area at 196 dpi within the thoracic segment (**A**), accompanied by a reduction of thoracic white matter area at 196 dpi (**B**) and a reduction of the area of rostral reticulospinal tract (rrts) within the thoracic segment at 98 dpi and within the cervical and thoracic segment at 196 dpi (**C**). Graphs display box and whisker plots. Significant differences between groups detected by Mann–Whitney *U*-test were indicated by asterisks (* *p* ≤ 0.05), GM = gray matter, WM = white matter.

Area quantification of spinal cord segments revealed a significant reduction of cross-sectional area (CSA) in the thoracic segment of TMEV infected mice at 196 dpi (Figure 2A) associated with a reduction of white matter (WM) (Figure 2B) and rostral reticulospinal tract (rrts; Figure 2C) within the thoracic segment at the same time point. Furthermore, rrts area was significantly reduced within the thoracic segment at 98 dpi as well as in the cervical segment at 196 dpi (Figure 2C). 

### 2.3. Inflammation and Demyelination

**Figure 3:**
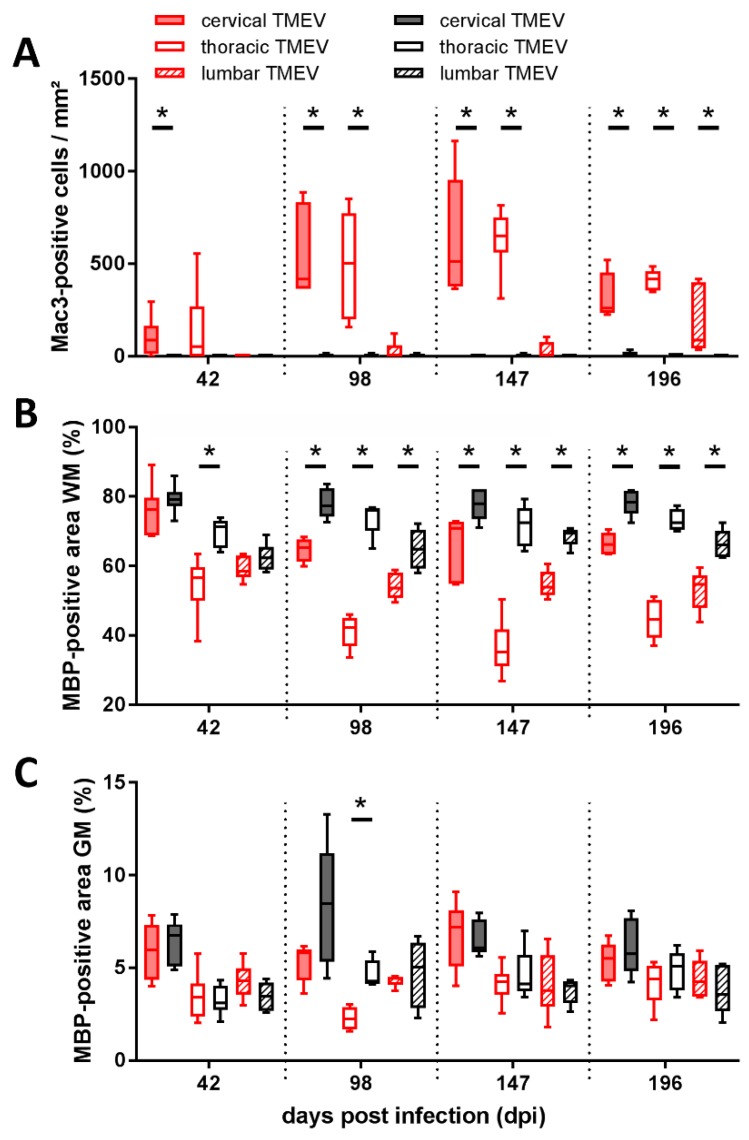
Evaluation of infiltrating macrophages within the area of the rostral reticulospinal tract revealed a significant increase in Mac3^+^ cells within the cervical segment at 42 dpi, within the cervical and thoracic segment at 98 and 147 dpi as well as within all investigated segments at 196 dpi (**A**). Assessment of myelin basic protein (MBP) stained slices revealed a significantly reduced MBP positive white matter (WM) area in the thoracic segment at 42 dpi and in all investigated segments at 98, 147, and 196 dpi (**B**). Moreover, the thoracic segment showed a significant reduction of MBP positive area in the gray matter (GM) at 98 dpi (**C**). No significant differences between TMEV-infected groups comparing 147 and 196 dpi were detected. Graphs display box and whisker plots. Significant differences between groups detected by Mann–Whitney *U*-test were indicated by asterisks (* *p* ≤ 0.05).

The density of microglia/macrophages was significantly increased within the cervical segment of TMEV-infected mice at 42 dpi (Figure 3A). Progressive caudal dissemination of inflammation was present and the density of microglia/macrophages was significantly increased within the cervical and thoracic SC segment at 98 dpi and 147 dpi and within all three investigated segments at 196 dpi (Figure 3A). Significant WM demyelination was detected within the thoracic segment at 42 dpi and in all segments at 98 dpi, 147 dpi, and 196 dpi (Figure 3B). Gray matter (GM) demyelination was present within the thoracic segment at 98 dpi (Figure 3C). 

### 2.4. Axonal Density

**Figure 4:**
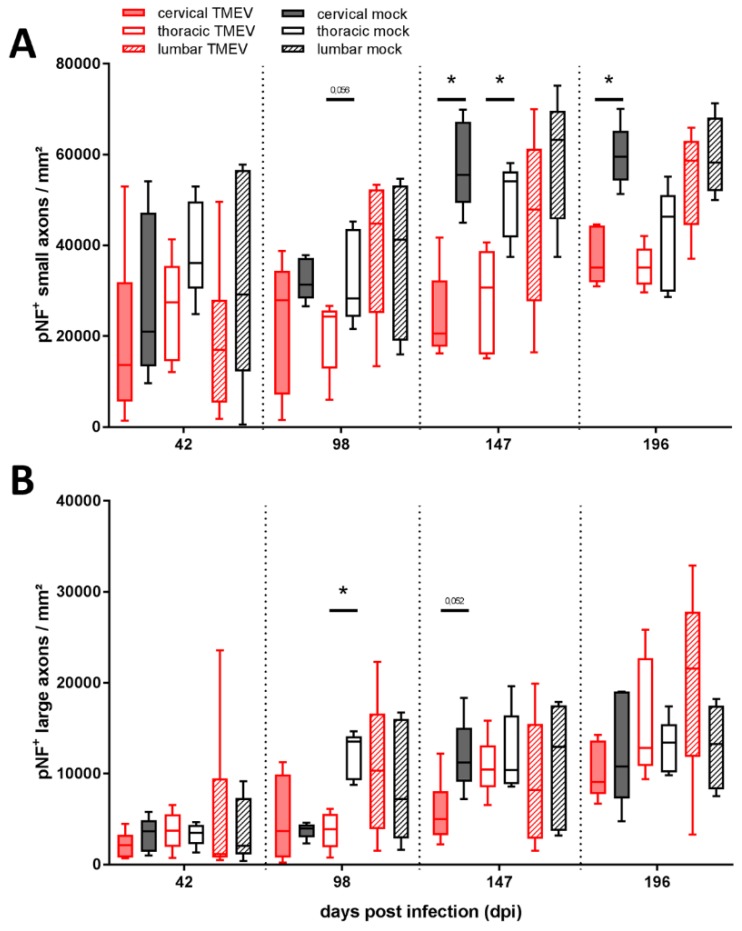
Evaluation of axonal loss showed a decreased number of phosphorylated neurofilaments (pNF) positive small (1–4<μm) diameter axons within the thoracic and cervical spinal cord at 147 dpi and within the cervical segment at 196 dpi (**A**). Large (≥4 μm) axons were significantly decreased within the thoracic segment at 98 dpi (**B**). No significant differences between TMEV-infected groups comparing 147 and 196 dpi were detected. Graphs display box and whisker plots. Significant differences between groups detected by Mann–Whitney U-test were indicated by asterisks (* *p* ≤ 0.05).

Immunohistochemistry targeting phosphorylated neurofilaments (pNF) revealed a significant reduction of small axons in the cervical segment at 147 and 196 dpi as well as in the thoracic segment at 147 dpi (Figure 4A). A loss of large axons was detected in the thoracic segment at 98 dpi (Figure 4B). Furthermore, a trend towards reduction of small fibers within thoracic segment at 98 dpi (*p* = 0.056) and large fibers in the cervical segment at 147 dpi (*p* = 0.052) was detected.

### 2.5. Ultrastructural Investigation of Myelin and Axons

**Figure 5:**
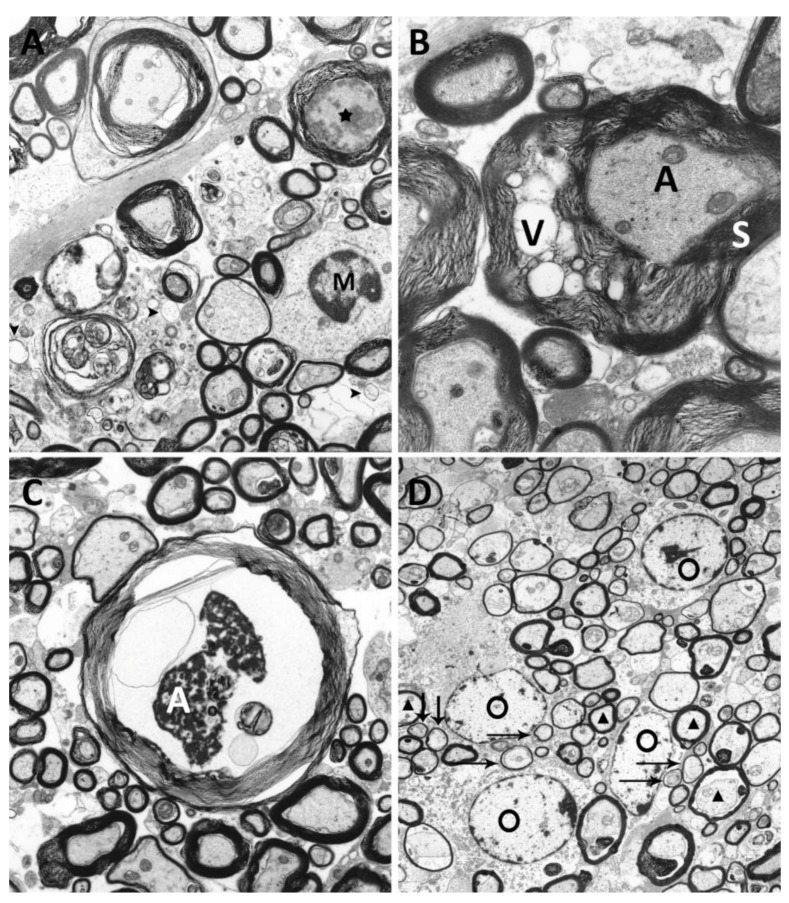
Ultrastructural analysis of degenerative changes of axons and myelin sheaths of TMEV infected SJL-mice. (**A**) Demyelinated area with denuded axons [arrowheads] and infiltration of microglia/macrophages [M] and axonal degeneration [asterisk] within the ventrolateral areas of the spinal cord (TMEV infected animal, 42 dpi, magnification 5000×). (**B**) Vacuolation [V] of myelin sheath [S] surrounding an intact axon [A] (TMEV infected animal, 98 dpi, magnification 10,000×). (**C**) Axonal [A] degeneration characterized by shrinkage, an uneven axonal membrane and accumulation of electron dense material (TMEV infected animal, 98 dpi, magnification 5000×). (**D**) Multiple remyelinated axons [arrows], characterized by thinner myelin sheaths compared to normally myelinated fibers [triangles], characteristic for oligodendrocyte [O] mediated remyelination were detected (TMEV infected animal, 196 dpi, magnification 6300×).

Mock infected animals revealed no ultrastructural alterations at all investigated time points. Within TMEV infected animals, infiltration of macrophages, completely denuded axons (Figure 5A), degenerative myelin changes, characterized by vacuolation of myelin sheaths (Figure 5B), axonal degeneration (Figure 5C), as well as remyelination (Figure 5D) were detected. TMEV infected animals showed axonal alterations at all investigated time points. Degenerative changes affected 2.2% (42 dpi), 4.5% (98 dpi), 2.8% (147 dpi), and 1.8% (196 dpi) of all investigated axons. As described previously within the same animal cohort [40] vacuolation of myelin sheaths (2.2%), and complete demyelination (5.8%) of investigated axons were observed at 42 dpi. The degree of demyelination increased until 98 dpi, where 2.8% of axons showed vacuolated myelin sheaths and 8.4% of axons revealed complete myelin loss. Thereafter, the percentage of vacuolated myelin sheaths decreased to 0.2% at 147 dpi and 0.5% at 196 dpi. Moreover, the percentage of completely demyelinated fibers also declined, with 6.3% at 147 dpi and 5.0% at 196 dpi. Microglia/macrophages, partly with intracytoplasmic myelin components (myelinophages) were present at all investigated time points. Remyelination was firstly detected at 147 dpi (0.5% of investigated axons) and increased until 196 dpi (3.2% of investigated axons).

## 3. Discussion

TMEV-IDD is a virus-mediated animal model particularly mimicking features of the progressive forms of MS [27,28,41]. Spinal cord lesions in TMEV-IDD are predominantly located within the ventral and lateral aspects of the thoracic spinal cord [39,42,43,44]. Within TMEV-IDD and MS, spinal cord lesions are assumed to play a key role in mediating clinical disability [14]. The present study firstly compares histopathological findings in the SC in TMEV-IDD with recently published SC lesions in progressive MS [17]. 

The progression of clinical disability as well as histopathological and immunohistochemical lesions in TMEV-IDD and MS show many similarities but also differences (Table 1). In TMEV-IDD, animals showed a continuous deterioration of motor coordination and performance, starting at 63 dpi and clinical scores were persistently elevated following 91 dpi. Earlier detection of clinical disability in TMEV-infected mice using rotarod compared to clinical scoring may be attributed to a higher sensitivity of the rotarod test. However, the onset of clinical impairment after 63 dpi represents a consistent finding in TMEV-IDD [42,45,46]. All MS patients included in the study showed severe clinical impairment consistent with an expanded disability status scale of ≥7 [17].

TMEV-infected animals showed early and most prominent lesions within the cervical and thoracic SC segments, which is in accordance with previous investigations in TMEV-IDD [28,39,42,43]. Similar to MS patients in the chronic phase, TMEV-infected animals showed most prominent reduction of investigated SC areas in the thoracic, followed by the cervical segment. A similar spatial distribution was also observed regarding neuronal loss within the GM of SC [47]. 

Reduction of thoracic CSA in TMEV-infected mice at 196 dpi was accompanied by a general reduction of WM and a decreased rrts area. No significant GM involvement was detected at this time point. Therefore, it can be assumed that WM changes are particularly responsible for reduction of the thoracic CSA. In MS, there is a large variability in SCA within studies, most likely due to heterogenicity in study populations, distribution of lesions, anatomical localization of the evaluated spinal cord segment, and applied methods [14]. During TMEV-IDD variable results regarding SCA and related parameters dependent on the investigated SC segment and time span after infection with the Daniels strain (DA) of TMEV are described [29,31,32]. In the present study, CSA reduction was restricted to the thoracic spinal cord segment at one time point. However, other TMEV studies indicate that SCA may represent an important feature in TMEV-IDD pathogenesis at later time points [29] and it cannot be excluded that CSA atrophy might be obscured by an inflammation-dependent intraspinal edema. 

One important difference between MS and TMEV-IDD is the lack of inflammatory infiltrates in chronic MS spinal cord lesions [17]. Inflammation within the ventromedial WM of TMEV-infected mice showed a rostro-caudal spread. The timely dependent, spatial distribution of inflammation in TMEV-IDD perfectly mirrors the spread of TMEV and demyelination [42,48,49]. Interestingly, inflammation and demyelination in TMEV-IDD was not correlated with SCA, WM reduction, or axonal loss in rrts. Although inflammation is present in all stages of MS, it declines over time especially in the progressive phase of the disease, moving the focus to potential neurodegenerative processes [50,51,52,53]. In progressive MS, activated microglia/macrophages are thought to be an important mediator of mitochondrial damage in axons [53]. In advanced TMEV-IDD SC lesions inflammatory cells consist of microglia/macrophages, T-lymphocytes, and lower numbers of B-lymphocytes [42,48,49].

The question whether axonal loss and atrophy occur as a result of neuroinflammation, as stated in the outside-in model, or that axonal damage itself acts as trigger for consequent inflammatory reactions, as postulated in the inside-out model, still remains to be clarified, especially in progressive MS, while both mechanisms have been shown to contribute to TMEV-IDD pathogenesis [33,50,54,55,56]. Moreover, all investigated spinal cord segments of infected mice revealed significant white matter demyelination (WMD) at 98, 147, and 196 dpi. Microglia/macrophages, which are crucially involved in viral persistence in TME, are closely linked to WMD [40,57,58]. WMD in the SC represents one of the prominent features of TMEV-IDD, whereas in MS, especially GM atrophy in the SC is thought to play a crucial role [52,59,60]. Significant demyelination in SJL mice preceded detectable changes in CSA and axonal density in the present study. These findings suggest that axonal damage might be a consequence of inflammation and demyelination [32,37]. Nevertheless, several studies postulated that SCA was more likely related to axonal loss than demyelination, and that axonal damage even preceded demyelination in TMEV-infected animals [31,61,62]. Ultrastructural analysis in TMEV-IDD revealed degenerative changes affecting myelin and axons at all investigated time points, substantiating and detailing the immunohistochemical findings. Hallmarks in MS and TMEV-IDD are demyelination and axonal loss. In the present study, demyelination preceded axonal loss concomitant with reduction of rrts. This finding indicates that demyelination alone does not sufficiently explain the reduction of SC volume. Furthermore, it underlines a potential high relevance of axonal loss in descending motor pathways regarding chronic impairment. 

Studies investigating SC lesions in MS reveal contrasting results regarding the predominantly affected axon diameters [17,31,32,38,55,63,64,65]. Similar results are obtained in TMEV-IDD. The present study using the BeAn strain of TMEV identified a preferential loss of small diameter axons within the rrts starting at 98 dpi in the thoracic spinal cord segment extending to the cervical segment at the following time points. This contrasts with studies using the DA strain of TMEV which identified a predominant loss of large diameter axons [31,32,66]. Pathomechanisms detailing the selectiveness of TMEV strains for small versus large diameter axons are currently unknown. Factors contributing to different selectivities include virus strain, spatial localization, as well as time point post infection. However, TMEV-IDD and SC lesions in MS have in common that progressive clinical disability is accompanied by axonal degeneration and loss with all axon diameters affected.

## 4. Materials and Methods

### 4.1. Animals and Tissue Processing

Murine spinal cord samples from a previous study were used for this investigation [49]. Groups of five to six, female SJL/JCrl mice were infected with 1.63 × 10^6^ plaque forming units/mouse of the BeAn strain of TMEV or cell culture supernatant into the right cerebral hemisphere at five weeks of age [27]. Clinical investigation and an accelerated rotarod performance test (TSE Systems GmbH, Bad Homburg, Germany) were performed weekly as previously described [49,67]. Rotation speed continuously increased from 5 to 55 rounds per minute (RPM) within 5 min. A mean value of three consecutive runs was used for statistical analysis. Experiments were terminated after 42, 98, 147 and 196 dpi and tissue samples were collected for further investigations. 

**Figure 6:**
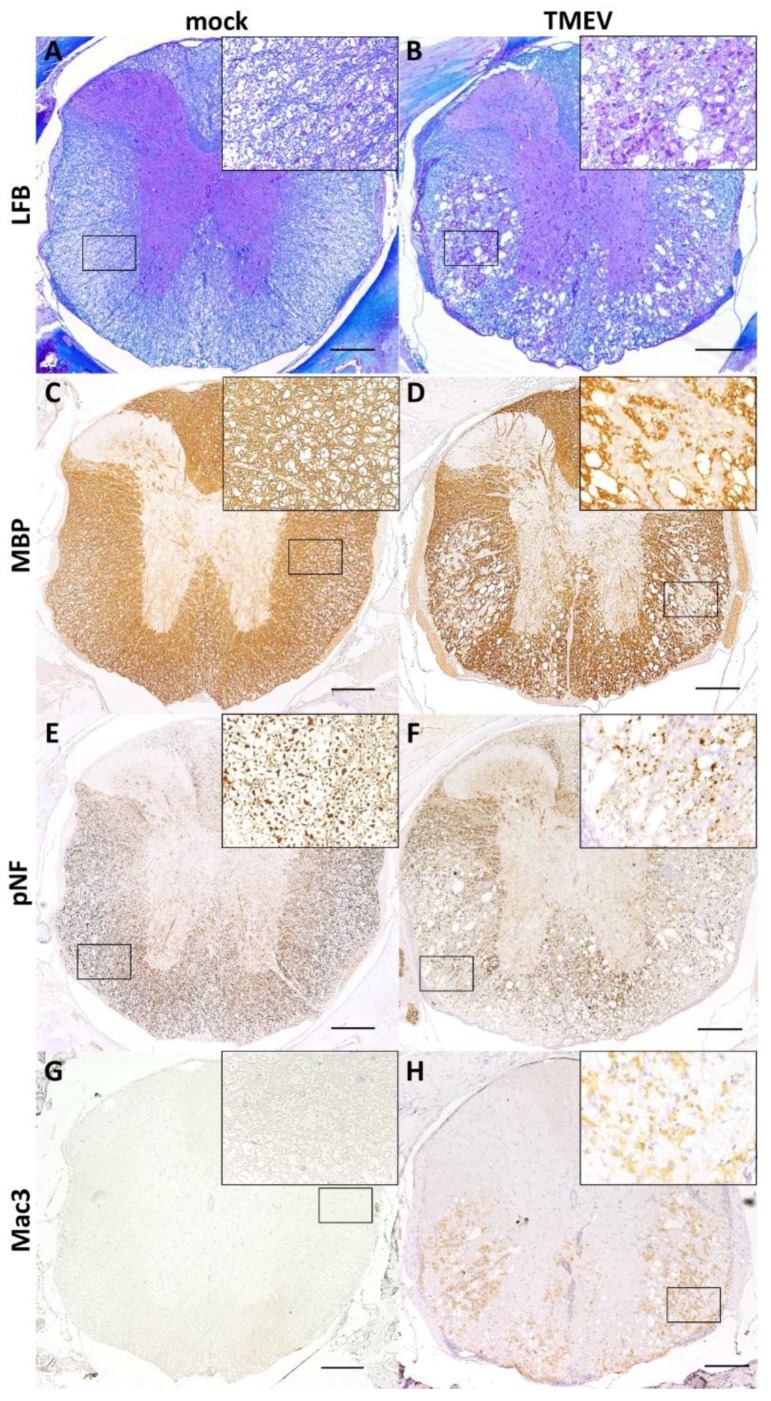
Histochemical and immunohistochemical evaluation of SC segments using Luxol fast blue (LFB) staining (**A**: mock infected animal showing regularly myelinated white matter; **B**: TMEV infected animal with multifocal areas of demyelination). Immunohistochemistry targeting myelin basic protein (MBP; **C**: mock infected animal showing regularly myelinated white matter; **D**: TMEV infected animal with multifocal demyelination), phosphorylated neurofilaments (pNF; **E**: mock infected animal showing regularly distributed pNF labeled axons; **F**: TMEV infected animal with multifocal loss of pNF labeled axons) and microglia/ macrophages (Mac3; **G**: mock infected animal showing no immunolabeled cells; **H**: TMEV infected animal revealing multifocal immunolabeled cells within the white matter). Pictures show the thoracic segment of a mock (clinical score: 0, rotarod: 27.8 rpm) and a TMEV-infected animal (clinical score: 4; rotarod: 14.5 rpm) at 98 dpi. Scale bars = 200 μm. Inserts in 400× magnification.

Cervical (C1–C3), thoracic (Th3–Th7) and lumbar (L1–L2) spinal cord segments within their vertebral bodies were fixed in 10% neutrally buffered formalin and subsequently decalcified for 48 h using a 10% disodium-ethylenediaminetetraacetate solution. Tissue was embedded in paraffin wax, cut on a microtome and subsequently stained with HE and/or LFB for localization of inflammatory and demyelinating lesions (Figure 6A,B). For further evaluation, immunohistochemistry detecting myelin basic protein (MBP; AB980; polyclonal; Chemicon, Temecula, CA, USA; 1:500; Figure 6C,D), phosphorylated neurofilaments (pNF; SMI-312R; monoclonal; Sternberger Monoclonals, Lutherville, Maryland, USA; 1:8000; Figure 6E,F) and macrophages/microglia (Mac3/CD107b; MCA2293; polyclonal; Bio-Rad, Hercules, CA, USA; 1:400; Figure 6G,H) in combination with the avidin-biotin-peroxidase complex (ABC) method (Vector Laboratories, Burlingame, CA, USA; MBP, Mac3) or the EnVision+ System-HRP (Dako; Hamburg, Germany; pNF) were used on spinal cord cross sections.

### 4.2. Area Measurements

**Figure 7:**
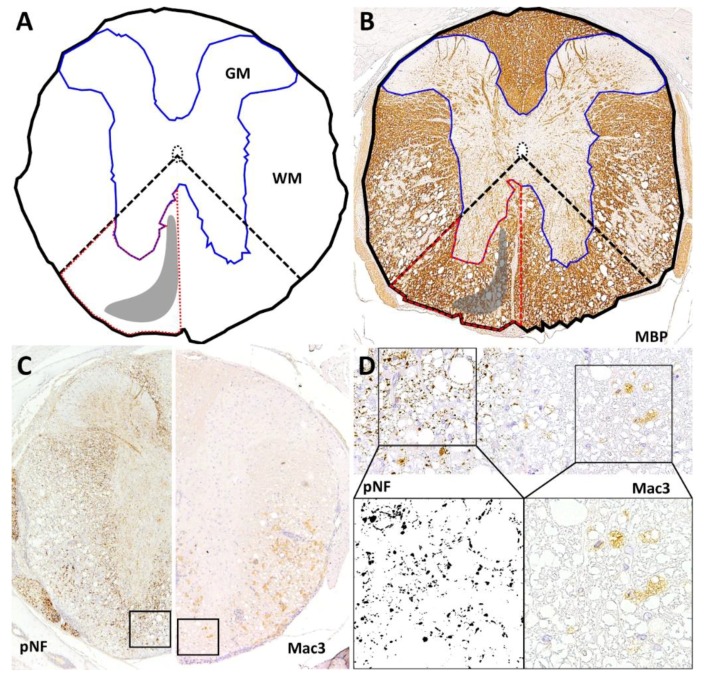
Quantification of cross-sectional area (CSA; black line), white matter (WM), gray matter (GM; blue line) and the approximated area of the rostral reticulospinal tract (rrts; red dashed line) using MBP-stained sections. Localization of rrts is marked as gray area. Area of rrts was approximated via an angle bisector (dashed line) between a vertical and horizontal line intersecting at the central canal (matrix: **A**; application example: **B**) For assessment of axonal loss and inflammation, images from the rrts were taken at high magnification (40× objective). One picture was taken from the left and right ventromedial white matter each, saved as tiff file (**C**) and a counting frame (120 μm × 120 μm) was inserted (**D**). For quantification of axonal density, images were converted to 8bit grayscale (**D**). After an adjustment of threshold axons were counted and separated in small (1–<4 μm^2^) and large axons (≥4 μm^2^) via adjustment of size of the detected particles. Axonal density was calculated as pNF^+^ particles per mm^2^. Mac3^+^ cells within the counting frame were counted manually and calculated as Mac3^+^ cells per mm^2^ (**D**).

MBP-stained slides were digitalized by a DP72 camera (Olympus, Münster, Germany) mounted on a BX51 microscope (Olympus, Münster, Germany) using a 4× objective and saved as tiff files. The CSA, WM and GM, as well as the area of the left and right rrts were outlined manually using analySIS^®^ 3.2 software (SOFT Imaging System; Olympus, Münster, Germany) (Figure 7A,B). The area of rrts was approximated outlining the ventral white matter area in between the ventral median fissure and an angle bisector between a vertical and horizontal line intersecting at the central canal. 

The rrts represents an ipsilateral descending tract which is located adjacent to the ventral and ventromedial periphery of the ventral funiculus, keeping this position over the different SC segments [68,69]. In mammals, amongst other things such as modulation of sensory and autonomic functions, the projections of the reticulospinal tracts are involved in regulation of movement and posture [69,70].

### 4.3. Quantification of Inflammation

Microglia/macrophages were quantified on cervical, thoracic and lumbar spinal cord cross section using antibodies targeting Mac3. One picture was taken from the left and right ventromedial white matter and saved as tiff file (Figure 7C). A counting frame (120 µm × 120 µm) was inserted into each image, and positive cells were counted within this area (Figure 7D). Cell densities were calculated as Mac3-positive cells per mm^2^.

### 4.4. Demyelination

For evaluation of demyelination (loss of MBP-positive area), digitalized MBP-stained sections of cervical, thoracic and lumbar spinal cord were analyzed using analySIS^®^ 3.2 software (SOFT Imaging System; Olympus, Münster, Germany). Regions of interest (ROI; WM and GM) were manually outlined, and a threshold value adjusted. MBP-positive areas are provided as the percentage of investigated ROI. 

### 4.5. Axonal Density and Diameter

Evaluation of axonal density and average axonal diameter was conducted using anti-pNF stained spinal cord cross sections. Analogous to the procedure described above, two images from the ventromedial white matter were taken at high magnification and saved as tiff files (Figure 7C). Counting frames were inserted and images cut to the frame line. All images were duplicated for visual control of threshold setting and converted to 8bit grayscale using ImageJ (version 1.51q; http://imagej.nih.gov.ij/). Axons were detected using an adjustment of threshold (Figure 7D) followed by counting of axons via the “analyze particles” application. Axon counts were separated in small (1–<4 µm^2^) and large axons (≥4 µm^2^) modified from [66].

### 4.6. Ultrastructural Analysis

Cervical spinal cord segments of TMEV (BeAn strain, 1.63–10^6^ plaque forming units/mouse) and mock infected SJL/JHanHsd mice (5–6 animals per time point) were investigated using transmission electron microscopy as previously described [40,71]. Tissue samples were taken at 42, 98, 147 and 196 dpi. For each animal 100 axons including myelin sheaths located in the ventromedial area of the cervical spinal cord were investigated for myelin and axonal alterations. Myelin degeneration was characterized by vacuolation and complete loss of myelin sheaths. Axonal lesions were defined by swelling or shrinkage of fibers, an even or uneven axonal membrane, a marked accumulation of electron dense structures (dense bodies), a proliferation as well as swelling of mitochondria, as well as the presence of various abnormal organelles within the axoplasm and a proliferation of neurofilaments. Remyelinated axons were identified by a thinner myelin sheath compared to normally myelinated fibers.

### 4.7. Statistical Analysis

Statistical analysis was performed using SPSS for Windows (version 25; IBM^®^ SPSS^®^ Statistics, SPSS Inc., Chicago, IL, United States) using Kolmogorov-Smirnov and Mann–Whitney-U tests or two-way repeated measure ANOVA with post-hoc independent t-tests. Statistical significance was accepted at a *p*-value of ≤0.05. 

### 4.8. Ethics Statement

All animal experiments were conducted in accordance with the German Animal Welfare Law and were approved by the local authorities (Niedersächsisches Landesamt für Verbraucherschutz und Lebensmittelsicherheit (LAVES), Oldenburg, Germany, permission numbers: 33.9-42502-04-07/1331 and 33-42502-05/963).

## 5. Conclusions

The morphological changes during TMEV-IDD reflect the changes of progressive MS forms with respect to demyelination, SCA, and axonal loss. With regard to the rostro-caudal spatial localization, similarities between lesion sites in TMEV-IDD and MS spinal cord lesions were observed. However, demyelination in MS mainly affects GM while in TMEV-IDD predominantly WM is affected. Studies investigating SC lesions in MS show that axons of all diameters are affected, which is in concordance with findings in TMEV-IDD. However, in TMEV-IDD the virus strain and/or the localization within the spinal cord seem to have an impact upon the predominantly affected axon diameter. In TMEV-IDD SCA was restricted to the thoracic segment at 196 dpi, which may indicate that SCA is not a predominant finding in TMEV-IDD or may occur at later time points. The results of this comparative investigation further highlight the suitability of TMEV-IDD as animal model for the investigation of progressive MS.

## Figures and Tables

**Table 1:** Direct comparison between Theiler’s murine encephalomyelitis virus induced demyelinating disease (TMEV-IDD) and secondary progressive multiple sclerosis (MS) spinal cord (SC) lesions.

	Parameter	MS (Petrova et al. 2018)	TME
**Material**		whole post mortem spinal cords	cervical, thoracic and lumbar spinal cord segments (42, 98, 147, 196 dpi);
		people with secondary progressive MS and control subjects	TMEV and mock infected SJL mice
**Area measurement (Figure 2)**	decreased CSA	thoracic > cervical	thoracic segment, 196 dpi
	decreased aCST/rrts	cervical > thoracic	thoracic segment 98, 196 dpi; cervical segment 196 dpi
	GM reduction;	cervical > lumbar	-
	WM reduction	cervical > thoracic	thoracic segment, 196 dpi
**Inflammation (Figure 3)**	CD68/Mac3	no difference between lesions and non-lesional MS tissue	significant increase of microglia/ macrophages with rostro-caudal dissemination of inflammation in TMEV-infected animals
**Axonal loss (Figure 4)**	axonal density	significantly reduced in MS patients	significantly reduced at 98, 147 and 196 dpi in TMEV infected animals
	Level	all levels equally affected	cervical (147, 196 dpi) and thoracic (98, 147 dpi) segments
	axonal size	no difference between large and small diameter axons	large fibers (98 dpi); small fibers (147, 196 dpi)
**Demyelination (Figure 3)**	GM	more extensive (24%–48%) most severe within the thoracic segment	less extensive; thoracic segment, 98 dpi
	WM	less extensive (11%–13%); no variation in cord levels	more extensive; cervical segment, 42 dpi; all three segments, 98, 147, 196 dpi

MS = multiple sclerosis; TMEV = Theiler’s murine encephalomyelitis virus; CSA = cross sectional area; aCST = area of corticospinal tract; rrts = rostral reticulospinal tract; GM = gray matter; WM = white matter.

## Abbreviations

ABC	Avidin-biotin-peroxidase complex method
aCST	Area of corticospinal tract
CNS	Central nervous system
CSA	Cross sectional area
DA	Daniels strain
Dpi	Days post infection
GM	Grey matter
MBP	Myelin basic protein
MRI	Magnetic resonance imaging
MS	Multiple sclerosis
NAWM	Normal appearing white matter
pNF	Phosphorylated neurofilaments
PPMS	Primary progressive multiple sclerosis
ROI	Regions of interest
RPM	Rounds per minute
RRMS	Relapsing remitting multiple sclerosis
rrts	Rostral reticulospinal tract
SC	Spinal cord
SCA	Spinal cord atrophy
SPMS	Secondary progressive multiple sclerosis
TMEV	Theiler’s murine encephalomyelitis virus
TMEV-IDD	Theiler’s murine encephalomyelitis induced demyelinating disease
WM	White matter
WMD	White matter demyelination
